# Detection of Latent Tuberculosis in Chronic Kidney Disease (CKD) Patients on Dialysis Using the Cy-TB Test: A Hospital-Based Cross-Sectional Study

**DOI:** 10.7759/cureus.103636

**Published:** 2026-02-15

**Authors:** Harikrishnan Jagadeesan, Anuradha Hariharan, Sujatha Sathananthan, Manimala Manivannan, Arunagiri Gunasekar, Mugiya Thavasi

**Affiliations:** 1 Department of General Medicine, Government Chengalpattu Medical College and Hospital, Chennai, IND; 2 Department of General Medicine, Government Medical College and Hospital, Thiruvallur, Chennai, IND; 3 Department of Community Medicine, Government Medical College, Ariyalur, Ariyalur, IND; 4 Department of TB and Respiratory Medicine, Government Medical College and Hospital, Thiruvallur, Chennai, IND; 5 Department of Internal Medicine, Government Medical College and Hospital, Thiruvallur, Chennai, IND; 6 Department of Medical Education, Government Medical College and Hospital, Thiruvallur, Chennai, IND

**Keywords:** chronic kidney disease (ckd), cy-tb, latent tuberculosis infection, maintenance hemodialysis, tb prevention

## Abstract

Background

Latent tuberculosis infection (LTBI) represents a major health burden in chronic kidney disease (CKD) patients on hemodialysis due to severe immunosuppression. Current diagnostic methods have limitations in this population. The Cy-TB test is a novel immunological assay for detecting TB infection, but its performance in patients with CKD undergoing dialysis is unknown.

Objectives

The primary objective was to estimate the prevalence of LTBI in CKD patients on maintenance hemodialysis using the Cy‑TB skin test and to describe its agreement with interferon‑gamma release assay (IGRA) among Cy‑TB-positive cases. Secondary objectives were to identify clinical and demographic factors associated with Cy‑TB positivity and to explore whether the early dialysis phase (<6 months) represents a higher‑yield window for LTBI screening.

Methods

This hospital-based cross-sectional study enrolled 95 consecutive CKD patients on maintenance hemodialysis. All participants underwent Cy-TB testing with induration measurement at 48-72 hours; positive cases were confirmed by IGRA reference testing. Clinical and demographic data were collected through structured assessment. Diagnostic accuracy metrics, odds ratios (ORs) with 95% confidence intervals (CIs), and effect sizes (Cohen's d) were calculated using logistic regression and univariable statistical analysis.

Results

The overall prevalence of LTBI was 4.2% (4/95; 95% CI: 1.2%-10.3%), substantially lower than the 15-25% range reported in prior international dialysis cohorts. Dialysis duration <6 months emerged as the strongest independent predictor of Cy-TB positivity (p=0.001; OR=13.5; 95% CI: 2.5-72.8), with 75% of positive cases (3/4) occurring within this early dialysis phase and a 17.6% prevalence compared to 1.3% in chronic dialysis patients (≥6 months duration). Prior history of tuberculosis was significantly associated with increased positivity risk (p=0.048; OR=25.2; 95% CI: 1.4-442.8). Urban residence (p=0.073), presence of comorbidities (p=0.072), and elevated fever (p=0.099) showed marginal associations. Age, gender, body mass index, smoking status, alcohol consumption, TB contact history, and respiratory symptoms demonstrated no significant associations. Among the four LTBI‑positive cases identified, the Cy‑TB test showed perfect agreement with IGRA; however, this finding is based on a very small number of events and should be interpreted cautiously.

Conclusions

In this single-center cross-sectional cohort, the Cy-TB test showed excellent agreement with IGRA for LTBI detection in CKD-dialysis patients, based on a small number of LTBI-positive cases. The observed association between the early dialysis phase (<6 months) and higher LTBI detection suggests this period as a potential priority window for LTBI screening. These findings are exploratory and hypothesis-generating and require confirmation in larger, comparative, and longitudinal studies before any changes to screening strategies are recommended.

## Introduction

Tuberculosis (TB) remains one of the most significant global health threats, with the World Health Organization reporting 10.7 million new TB cases and 1.23 million TB-related deaths in 2024. Despite decades of TB control efforts, the global TB burden has reversed its declining trend, with TB incidence increasing from 10.7 million in 2022 to 10.8 million in 2023, representing only an 8.3% reduction since 2015, far short of the 50% reduction target established by the WHO End TB Strategy for 2025 [1]. India alone carries 26% of the global TB burden, with an estimated 2.7 million TB cases reported in 2023 and 320,000 TB-related deaths annually, the highest burden of any country worldwide. While India has achieved an 18% decline in TB incidence from 2015 to 2023, accelerated efforts are urgently needed to achieve the national target of TB elimination by 2025 [2,3].

Latent tuberculosis infection (LTBI), affecting approximately one-quarter of the world's population or 1.7 billion people, represents a critical yet often overlooked component of the global TB burden [4]. While LTBI causes no clinical symptoms and is not transmissible, approximately 5-10% of cases progress to active TB disease during the patient's lifetime, with substantially higher progression rates in immunocompromised populations [5]. Early identification and treatment of LTBI through standard LTBI regimen therapy reduces progression to TB disease by approximately 60% and is a cornerstone of TB elimination strategies endorsed by the WHO [6].

The prevalence of LTBI in CKD-dialysis populations ranges from 20% to 70%, depending on the diagnostic method and geographic region, with studies from India documenting a high TB burden in dialysis patients and higher LTBI rates documented in other countries, including Turkey (32.1%) and Thailand (25.9%) [7,8]. Chronic kidney disease (CKD) patients on maintenance hemodialysis represent one of the most vulnerable populations with dramatically elevated TB risk. The uremic state in advanced CKD is characterized by profound immune dysfunction, including defective T-cell function, reduced interferon-gamma production, impaired B-cell responses, and dysregulation of innate immunity. Population-based studies have documented that TB incidence in dialysis patients reaches 25-fold higher rates than in the general population [7,9]. Approximately 80% of active TB disease in dialysis patients results from reactivation of LTBI acquired before or at dialysis initiation rather than recent TB exposure [9]. Moreover, TB in dialysis patients presents with atypical, often nonpulmonary manifestations, leading to delayed diagnosis and substantially worse clinical outcomes, with mortality hazard ratios of 4.7-6.1 compared with TB patients with normal renal function [10].

Despite the extraordinarily high TB burden in CKD-dialysis populations and the availability of effective TB preventive therapy, systematic LTBI screening in dialysis patients remains uncommon in many settings, including India. The primary barrier to TB prevention in this vulnerable population has been the inadequacy of current diagnostic tests for LTBI detection in severely immunocompromised patients. Traditional tuberculin skin testing (TST) suffers from poor sensitivity and specificity in dialysis patients due to uremia-induced T-cell anergy and reduced capacity to mount delayed-type hypersensitivity reactions. While interferon-gamma release assays (IGRAs) offer improved specificity compared with TST, their sensitivity declines significantly with prolonged dialysis duration, resulting in increasing rates of false-negative results in this population [11,12]. Furthermore, IGRA testing requires centralized laboratory infrastructure, blood collection systems, and trained personnel, making widespread implementation challenging in resource-limited settings.

To address these critical diagnostic gaps, the Cy-TB test has been developed by the Serum Institute of India as a next-generation LTBI diagnostic solution. The Cy-TB test is an *M. tuberculosis* antigen-based skin test employing TB-specific antigens (recombinant ESAT-6 and CFP-10) that are absent in BCG vaccine strains and most nontuberculous mycobacteria, providing superior specificity compared with traditional TST [13,14]. Results are read at 48-72 hours after intradermal administration, similar to the traditional TST, enabling point-of-care testing at community, clinic, and hospital settings without the requirement for complex laboratory infrastructure [13,14]. Initial evidence demonstrates that Cy-TB achieves diagnostic performance comparable to or superior to IGRA tests, with reported specificity comparable to IGRA reference standards [13,14]. This hospital‑based cross‑sectional study had a primary objective of estimating the prevalence of LTBI using the Cy‑TB test in CKD patients on maintenance hemodialysis and describing its agreement with IGRA among Cy‑TB-positive participants. The secondary objectives were (i) to identify clinical and demographic factors associated with Cy‑TB positivity and (ii) to explore whether the early dialysis phase (<6 months from initiation) represents a higher‑yield temporal window for LTBI screening in this population.

## Materials and methods

This was a hospital-based, cross-sectional descriptive study conducted at the Government Medical College and Hospital, Thiruvallur, Tamil Nadu, India, over six months from April 2025 to September 2025. The study was approved by the Institutional Ethics Committee of Government Medical College and Hospital, Thiruvallur (approval no. IEC/4/2022) and conducted in accordance with the Declaration of Helsinki and Good Clinical Practice guidelines. Written informed consent was obtained from all study participants before enrollment. The study focused on patients with CKD Stage 5 undergoing maintenance hemodialysis to assess the prevalence of LTBI using the novel Cy-TB immunological test. The study was designed to evaluate the diagnostic performance of the Cy-TB test in this vulnerable immunocompromised population and to identify clinical and demographic risk factors associated with LTBI positivity.

The study was conducted at the Hemodialysis Unit of Government Medical College and Hospital, Thiruvallur. The hemodialysis unit comprises 25 stations with an average monthly census of 85-120 patients, providing a suitable patient population for this cross-sectional assessment. Consecutive patients with CKD Stage 5 undergoing maintenance hemodialysis at the unit were screened for study eligibility according to predefined inclusion and exclusion criteria until the required sample size of 95 was reached.

**Figure 1:**
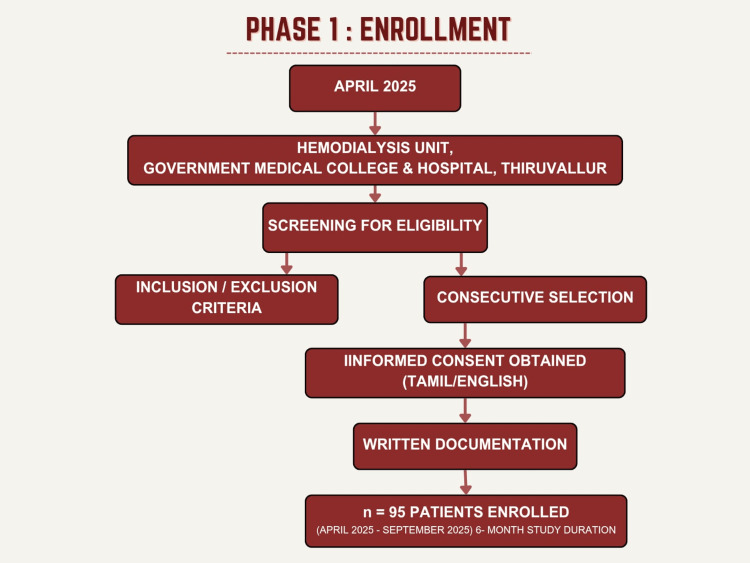
Enrollment of the participants

The sample size was estimated using the single‑proportion formula with a finite-population correction, based on an expected LTBI prevalence (p) of 30.2% in dialysis patients from Alemu et al. (2023) [15], a precision (d) of 0.05, and a 95% confidence level (z=1.96). For an infinite population, the initial sample size n_0_ was calculated as $n_0 = z^2 p(1-p) / d^2 \approx 323$. Applying the finite-population correction for an underlying dialysis population of N ≈ 120 at our center yielded a minimum required sample size of $n = \frac{n_0 N}{n_0 + N - 1} \approx 89$. We enrolled 95 participants to account for exclusions and ensure adequate precision. Figure 1 shows the flowchart for participant enrollment, which is Phase 1 of the study.

Patients were included in the study if they met all of the following criteria: (1) age 18 years or older at the time of enrollment; (2) confirmed diagnosis of CKD Stage 5, defined as estimated glomerular filtration rate (eGFR) less than 15 mL/min/1.73 m² using the Chronic Kidney Disease Epidemiology Collaboration (CKD-EPI) equation; (3) currently undergoing maintenance hemodialysis for a minimum of three months prior to study enrollment, ensuring that patients have been on dialysis long enough to reflect the chronic immunological changes associated with the uremic state; (4) hemodialysis frequency of at least twice weekly with each session lasting a minimum of four hours, consistent with standard dialysis regimens; (5) clinically stable status as determined by the treating nephrologist, indicating no acute complications or intercurrent illnesses requiring hospitalization at the time of enrollment; (6) willing and able to provide written informed consent in Tamil or English; and (7) absence of clinical signs or symptoms of active TB, including persistent cough lasting more than two weeks, documented fever (≥38.0°C), unintentional weight loss of 2 kg or more over the preceding three months, or night sweats causing sleep disruption.

Patients were excluded from the study if they had any of the following conditions or characteristics: (1) clinically unstable status, defined as presence of acute infection, hemodynamic instability, or uncontrolled comorbid conditions requiring intensive management; (2) unwillingness or inability to provide informed consent; (3) pre-existing skin conditions at the forearm injection site that could interfere with Cy-TB test administration or interpretation, such as eczema, psoriasis, dermatitis, extensive scarring, tattoos, or other dermatological conditions; (4) inability or unwillingness to return within 48-72 hours for test reading, as this time window is critical for accurate induration measurement; (5) documented history of active tuberculosis disease requiring anti-tuberculosis treatment or current receipt of anti-tuberculosis medications; (6) confirmed diagnosis of human immunodeficiency virus (HIV) infection, hepatitis B virus (HBV) infection with detectable HBsAg, or hepatitis C virus (HCV) infection, as these represent different immunosuppressive conditions requiring specific management; (7) active malignancy or other severe immunosuppressive conditions such as hematologic malignancies, systemic lupus erythematosus, or severe rheumatologic disease; and (8) documented history of solid organ transplantation or current use of long-term immunosuppressive medications including systemic corticosteroids at doses equivalent to more than 10 mg of prednisone daily, TNF-α inhibitors, other biological immunosuppressive agents, or chemotherapy medications.

**Figure 2:**
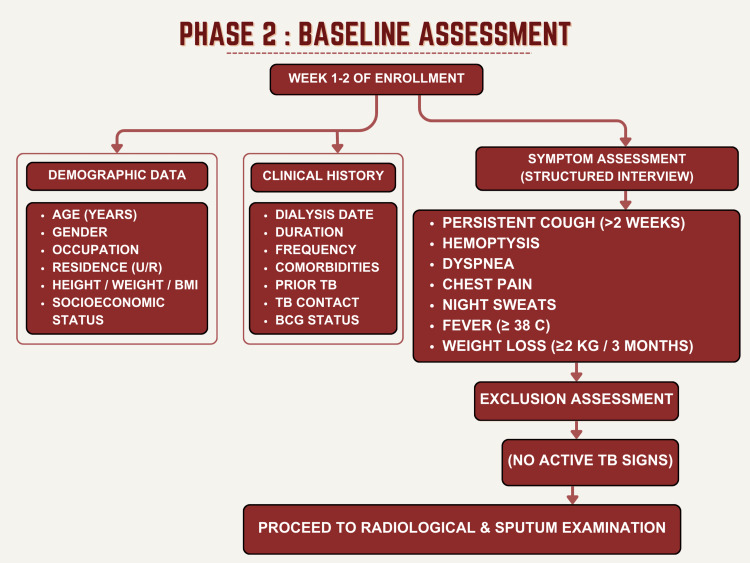
Baseline assessment of the participants

Following informed consent, comprehensive demographic and clinical data were collected. Data collected included age, gender, occupation, residence (urban/rural), anthropometric measurements (height, weight, BMI), underlying cause of CKD, hemodialysis parameters (duration, frequency, session length), TB-specific history (prior TB with treatment details, TB contact history within two years, BCG vaccination status), and presence of chronic comorbidities including diabetes mellitus, hypertension, bronchial asthma, and coronary artery disease. Symptom assessment in Figure 2, which is Phase 2 of the study, such as structured clinical interviews, documented respiratory symptoms (persistent cough >2 weeks, productive cough, dyspnea on exertion, hemoptysis, chest pain), and systemic symptoms (fever ≥38.0°C, night sweats, unintentional weight loss ≥2 kg over three months). These assessments were critical to exclude active TB disease.

**Figure 3:**
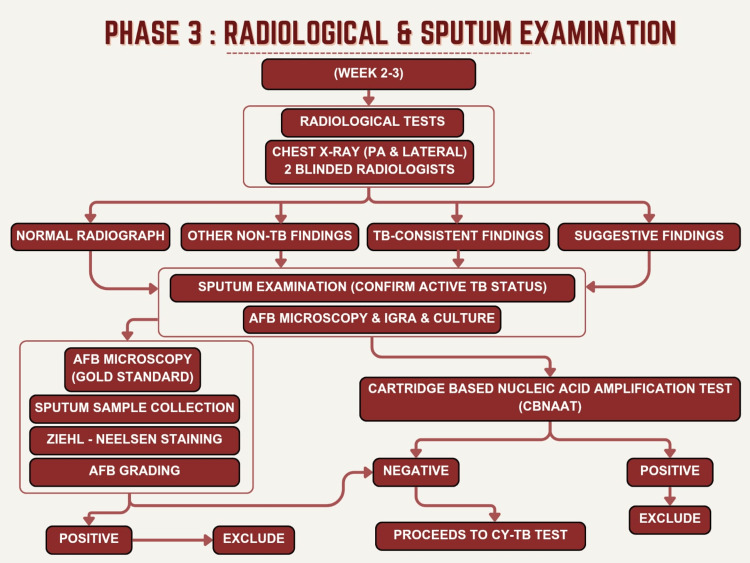
Radiological and sputum examination

Figure 3 depicts the radiological and sputum examination, which is Phase 3 of the study. All study participants underwent posteroanterior (PA) and lateral chest radiography using a standardized technique with patients in standard positioning and consistent radiation exposure parameters. Chest radiographs were independently reviewed by two experienced radiologists, each with at least 10 years of experience in pulmonary and thoracic radiology, who were blinded to the results of the Cy-TB test and all other study data. Sputum samples were obtained from all study participants to exclude active pulmonary TB, which was a key exclusion criterion. Participants were instructed on the proper sputum collection technique, emphasizing deep expectoration from the lower respiratory tract rather than saliva. All specimens were processed in the TB laboratory and examined for acid-fast bacilli (AFB) by smear microscopy by trained technicians, and cartridge-based nucleic acid amplification testing (CBNAAT) was performed to further exclude active TB disease.

**Figure 4:**
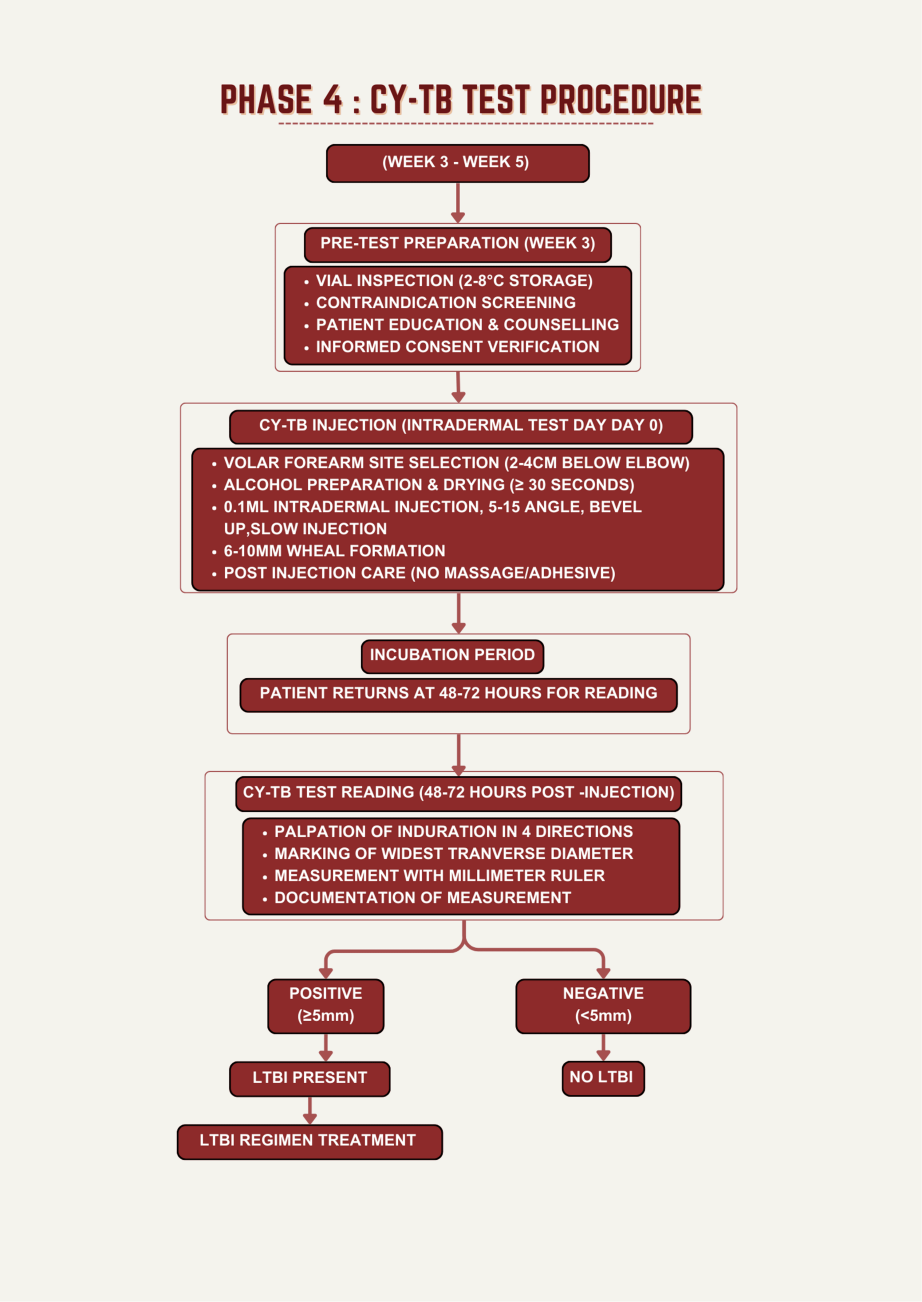
Cy-Tb test procedure

The Cy-TB test procedure and interpretation are presented in Figure 4, which is Phase 4 of the study. The Cy-TB test, an intradermal immunological test employing *Mycobacterium tuberculosis*-specific antigens (recombinant ESAT-6 and CFP-10), was administered according to National Tuberculosis Elimination Programme (NTEP) Standard Operating Procedures by certified healthcare workers [16]. Test vials (Serum Institute of India, Pune, India) containing 1 mL multi-dose antigen were stored at 2-8°C and used within the expiration date. After verification of participant identity and assessment for contraindications, 0.1 mL of Cy-TB solution was injected intradermally into the volar (palm-side) surface of the left forearm 2-4 cm below the elbow crease using strict aseptic technique with a tuberculin syringe and 26-gauge needle. The injection produced a characteristic 6-10 mm wheal; if absent, the injection was repeated on the opposite forearm. After injection, the site was not massaged, and participants received written instructions regarding site care.

The Cy-TB test reading was performed 48-72 hours after injection in a well-lit clinical setting by trained healthcare workers and updated in the NI-KSHAY (Ni=End, Kshay=TB), which is the web-enabled patient management system for TB control under the NTEP. It is developed and maintained by the Central TB Division (CTD), Ministry of Health and Family Welfare, Government of India, in collaboration with the National Informatics Centre (NIC) and the World Health Organisation Country Office for India.

With the patient's forearm slightly flexed, the injection site was gently palpated in four cardinal directions to locate induration borders. Only induration (firm, raised swelling) was measured; erythema and bruising were not recorded. The widest transverse diameter of induration perpendicular to the forearm's long axis was marked with a fine-point ballpoint pen and measured using a millimeter ruler to the nearest millimeter [16]. Cy‑TB readings were performed by trained healthcare workers according to NTEP standard operating procedures; however, formal blinding to participants’ clinical information and other study data was not implemented, which may introduce some risk of observer bias in border delineation and measurement. IGRA testing was performed only in participants with a positive Cy‑TB result as a confirmatory reference test; Cy‑TB‑negative participants were not routinely tested with IGRA. Consequently, formal diagnostic accuracy parameters for Cy‑TB, including sensitivity, specificity, and negative predictive value, could not be estimated. The present study, therefore, evaluates agreement between Cy‑TB and IGRA among Cy‑TB-positive cases, rather than full diagnostic accuracy in the entire cohort. Results were documented on standardized case report forms.

All data collected during the study were entered into a structured database using Microsoft Excel 2019 (Microsoft Corporation, Redmond, Washington), which included built-in validation rules and error-checking to minimize data entry errors. Double data entry was performed for 10% of randomly selected cases, and discrepancies were identified and corrected after review of the source documents. Periodic spot checks were conducted throughout the data entry process to verify consistency and accuracy. All patient-identifying information, including names, medical record numbers, and other personal identifiers, was replaced with unique study identifiers before analysis, and the link between study IDs and patient identities was maintained in a separate encrypted database with access restricted to the principal investigator only.

All statistical analyses were conducted using IBM SPSS Statistics for Windows, Version 25 (Released 2017; IBM Corp., Armonk, New York). Continuous variables were tested for normality with the Shapiro-Wilk test and summarized as mean ± standard deviation with range when normally distributed, or as median with interquartile range (25th-75th percentile) when non-normal. Categorical variables were summarized as counts (n) and percentages (%). The primary outcome, LTBI prevalence by Cy-TB, was calculated as the proportion of participants with Cy-TB positivity (induration ≥5 mm), with 95% confidence intervals derived using exact binomial methods. Between-group comparisons (Cy-TB-positive vs. negative) used independent-samples t-tests for normally distributed continuous variables, Mann-Whitney U tests for non-normal continuous variables, chi-square tests for categorical variables, and Fisher’s exact test when expected cell counts were <5, with two-tailed α=0.05. For association measures, crude odds ratios with 95% confidence intervals were estimated using logistic regression for categorical variables showing significant or borderline associations with LTBI. For continuous variables demonstrating significant between-group differences, Cohen’s d was calculated to quantify effect size, interpreted using standard thresholds (d<0.20 trivial, 0.20-0.50 small, 0.50-0.80 medium, >0.80 large). Analyses were conducted using a complete‑case approach; missing data, if present, were not imputed. Given the very small number of Cy‑TB-positive cases (n=4), all regression and subgroup analyses were prespecified as univariable and exploratory, and their results were interpreted with caution. Predefined subgroup analyses estimated LTBI prevalence across strata of dialysis duration (<6 months, 6-12 months, 1-2 years, >2 years), prior TB history, TB contact history, residence (urban/rural), comorbidity status, and socioeconomic status (low/middle/high), with chi-square tests comparing prevalence between subgroups. Data were entered into a structured Microsoft Excel 2019 database with embedded validation checks, and regular spot checks were conducted to ensure data quality. All identifiers were replaced with unique study codes before analysis, and the linkage file was stored separately in an encrypted, access-restricted database.

## Results

Study population and baseline characteristics

A total of 95 CKD patients on maintenance hemodialysis were enrolled in this cross-sectional study. The study population consisted of 57 males (60%) and 38 females (40%), with a mean age of 51.8±13.0 years (range: 23-73 years). The demographic and clinical characteristics of the study population are presented in Table 1. There were no significant differences in age, gender, or weight between Cy-TB-positive and negative groups. However, a highly significant difference was observed in dialysis duration, with positive cases having a substantially shorter duration of dialysis (3.75±2.87 months) compared to negative cases (38.4±30.1 months), representing a mean difference of 34.65 months (t=-3.347, df=93, p=0.001). The overall study population had a mean weight of 54.8±10.1 kg. Comorbidities were highly prevalent, with 68.4% (n=65) of patients having at least one comorbid condition. Hypertension was the most common comorbidity, affecting 57.9% (n=55) of participants, followed by diabetes mellitus in 31.6% (n=30) of patients. Notably, all four Cy-TB-positive cases (100%) had at least one comorbid condition, compared to 67% (n=61) of negative cases, representing a marginally significant trend (χ²=3.247, p=0.072).

**Table 1:** Demographic and Baseline Clinical Characteristics of the Study Population Data are presented as mean±SD or n (%). Continuous variables (age, weight, dialysis duration): Independent-samples t-test (two-tailed): Age: t=−0.186, df=93, p=0.854; Weight: t=−0.882, df=93, p=0.381; Dialysis duration: t=−3.347, df=93, p=0.001*. Categorical variables (gender, hypertension, diabetes, comorbidities): Chi-square test: Gender χ²=0.284, df=1, p=0.591; Hypertension χ²=1.348, df=1, p=0.244; Diabetes χ²=1.994, df=1, p=0.158; Any comorbidity χ²=3.247, df=1, p=0.072~. Significance levels: p<0.01 (highly significant); ~0.05<p<0.10 (marginally significant). Abbreviations: SD, standard deviation; n, number; df, degrees of freedom.

Characteristic	Positive (n=4)	Negative (n=91)	Total (n=95)	p-value
Age (years), mean±SD	50.0±11.4	51.9±13.1	51.8±13.0	0.854
Male, n (%)	3 (75)	54 (59)	57 (60)	0.591
Female, n (%)	1 (25)	37 (41)	38 (40)	-
Weight (kg), mean±SD	59.0±8.2	54.5±10.2	54.8±10.1	0.381
Dialysis duration (months), mean±SD	3.75±2.87	38.4±30.1	34.8±30.2	0.001*
Hypertension, n (%)	3 (75)	52 (57)	55 (58)	0.244
Diabetes mellitus, n (%)	2 (50)	28 (31)	30 (32)	0.158
Any comorbidity, n (%)	4 (100)	61 (67)	65 (68)	0.072~

Dialysis duration and LTBI detection

Dialysis duration was identified as the strongest predictor of Cy-TB positivity in this cohort. The independent-samples t-test revealed a highly significant difference between positive and negative cases (t=-3.347, p=0.001), with a large effect size (Cohen’s d=0.82). When stratified by duration categories, 75% (n=3) of positive cases occurred within the first six months of dialysis initiation. The prevalence of LTBI was dramatically higher in the <6 months group (17.6%, n=3/17) compared to the ≥6 months group (1.3%, n=1/78), representing a 13.5-fold difference in prevalence rates (χ²=12.456, df=3, p=0.006). Table 2 shows the LTBI prevalence stratified by dialysis duration category.

**Table 2:** LTBI Prevalence Stratified by Dialysis Duration Category Statistical test: Chi-square (χ²=12.456, df=3, p=0.006). Data: n, prevalence (%), 95% CI (exact binomial), p<0.01. Abbreviations: LTBI, latent tuberculosis infection; CI, confidence interval; df, degrees of freedom.

Dialysis Duration	Positive Cases	Negative Cases	Total	Prevalence Rate	95% CI
<6 months	3	14	17	17.6%	3.8–43.4%
6–12 months	0	8	8	0%	0–36.9%
1–2 years	1	24	25	4.0%	0.1–20.4%
>2 years	0	45	45	0%	0–8.0%
Total	4	91	95	4.2%	1.2–10.3%

Prior TB history and risk assessment

Prior history of TB was identified as a significant risk factor for LTBI detection in this study. Among the four Cy-TB-positive cases, one patient (25%) had a documented history of prior TB infection, compared to only one of 91 negative cases (1.1%). This difference was statistically significant (χ²=3.906, p=0.048). The odds ratio for LTBI positivity in patients with prior TB history was 25.2 (95% CI: 1.4-442.8), indicating a substantially elevated risk in this subgroup. The sensitivity of prior TB history for detecting LTBI was 25%, while its specificity was 98.9%, resulting in a positive predictive value of 50%.

Detailed profile of Cy-TB-positive cases

Four patients tested positive for LTBI by the Cy-TB test. Their detailed clinical and demographic profiles are presented in Table 3. The positive cases included three males (75%) and one female (25%). Patient 1 was a 51-year-old male who had been on dialysis for seven months at the time of testing, with an induration size of 12 mm, indicating mild-to-moderate reactivity. He had hypertension as a comorbidity but no prior history of tuberculosis. Patient 2 was a 54-year-old male with 24 months of dialysis exposure and an induration of 10 mm. Like Patient 1, he had hypertension but no prior TB history. Patient 3 was a 35-year-old female representing the youngest positive case, with only five months of dialysis exposure and the largest induration (20 mm). Notably, this patient had a documented history of TB and dual comorbidities (hypertension and diabetes). Patient 4 was a 60-year-old male with the shortest dialysis duration (three months) and the most robust Cy-TB response (induration 30 mm), with both hypertension and diabetes as comorbidities.

**Table 3:** Detailed Clinical and Demographic Profile of Cy-TB-Positive Cases (n=4) Individual case data presented as actual values; summary statistics as mean±SD, median, and range. Comparisons to the negative group: dialysis duration (t=-3.347, p=0.001); p<0.01. Abbreviations: HTN, hypertension; DM, diabetes mellitus; TB, tuberculosis; SES, socioeconomic status; M, male; F, female.

Case	Age	Gender	Dialysis Duration	Induration (mm)	HTN	DM	Prior TB	Urban	SES
1	51	M	7 months	12	Yes	No	No	Yes	Middle
2	54	M	24 months	10	Yes	No	No	Yes	Middle
3	35	F	5 months	20	Yes	Yes	Yes	Yes	Middle
4	60	M	3 months	30	Yes	Yes	No	Yes	High

The mean induration size among positive cases was 18±8.2 mm (median: 16 mm, range: 10-30 mm), with all values substantially exceeding the diagnostic threshold of 10 mm. The mean age of positive cases was 50±11.4 years (median: 52.5 years). Notably, all four positive cases (100%) were from urban areas, all four (100%) had at least one comorbid condition, and one (25%) had prior TB exposure. The mean dialysis duration for positive cases was 9.75±10.5 months (median: 6 months), which was significantly shorter than the 38.4±30.1 months in negative cases. Among the four LTBI‑positive participants, Cy‑TB and IGRA results were concordant in all cases, indicating high agreement within this small subgroup.

Risk factor analysis and odds ratios

A comprehensive chi-square analysis was performed to assess associations between demographic, clinical, and socioeconomic factors and Cy-TB positivity. Among all variables examined, prior TB history emerged as the only statistically significant risk factor (p=0.048, OR=25.2). Several variables showed marginally significant trends toward association with LTBI positivity, including urban residence (p=0.073), any comorbidity (p=0.072), fever or elevated temperature (p=0.099), monthly family income (p=0.087), and socioeconomic status category (p=0.092).

Notably, numerous clinical, demographic, and lifestyle variables showed no significant association with Cy-TB positivity. These included age (p=0.854), gender (p=0.591), body weight (p=0.381), smoking status (p=0.339), alcohol consumption (p=0.861), specific comorbidities (hypertension, p=0.244; diabetes, p=0.158), and respiratory symptoms including cough (p=0.403), dyspnea (p=0.359), productive cough (p=0.359), and night sweats (p=0.172). TB contact history (p=0.572) and BCG vaccination status (p=0.511) were also not significantly associated with positivity. Table 4 shows the risk factor analysis - odds ratios and 95% confidence intervals for LTBI association.

**Table 4:** Risk Factor Analysis - Odds Ratios and 95% Confidence Intervals for LTBI Association The statistical tests are the chi-square test and Fisher's exact test (expected cell frequencies <5). OR with 95% CI calculated by logistic regression. *p<0.05 are statistically significant; ~0.05<p<0.10 are marginal trends. Abbreviations: OR, odds ratio; CI, confidence interval.

Risk Factor	Cases (n)	Controls (n)	Odds Ratio	95% CI	p-value	Interpretation
Prior TB History	1	1	25.2	1.4–442.8	0.048	SIGNIFICANT
Urban Residence	4	50	∞	-	0.073~	Marginal trend
Fever/Elevated Temperature	1	5	6.3	0.6–64.3	0.099~	Marginal trend
Night Sweats	1	8	3.3	0.4–29.1	0.172	Not significant
Any Comorbidity	4	61	∞	-	0.072~	Marginal trend
Hypertension	3	52	2.2	0.4–12.1	0.244	Not significant
Diabetes Mellitus	2	28	2.2	0.4–12.1	0.158	Not significant
Smoking	0	24	-	-	0.339	Not significant
Cough	2	33	1.1	0.2–5.1	0.403	Not significant
Age (Continuous)	-	-	-	-	0.854	Not significant

Stratified prevalence rates and subgroup analysis

The prevalence of LTBI varied considerably across predefined subgroups, as shown in Table 5. When stratified by dialysis duration, the prevalence in the <6 months group (17.6%) was substantially higher than in the ≥6 months group (1.3%), representing a 13.5-fold difference. In subgroups defined by prior TB history, the prevalence among patients with prior TB was 50% (1/2), compared to 3.2% (3/93) in those without prior TB history. Urban residents demonstrated a higher prevalence (7.4%, 4/54) compared to rural residents (0%, 0/41), though this difference did not reach statistical significance. Patients with any comorbidity had a prevalence of 5.8% (4/69), compared to 0% (0/26) in those without comorbidity (p=0.072, marginally significant). When stratified by socioeconomic status, all positive cases were from the middle or high SES category (prevalence 6.9% in middle/high SES, 0% in low SES; p=0.092, marginally significant).

**Table 5:** Stratified Prevalence of LTBI in Clinically Relevant Subgroups Abbreviations: LTBI, latent tuberculosis infection; CI, confidence interval; TB, tuberculosis; SES, socioeconomic status.

Subgroup	Positive/Total	Prevalence (%)	95% CI	Comparison Group	Comparative Rate
Dialysis Duration	-	-	-	-	-
<6 months	3/17	17.6%	3.8–43.4%	≥6 months	1.3%
6–12 months	0/8	0%	0–36.9%	-	-
1–2 years	1/25	4.0%	0.1–20.4%	-	-
>2 years	0/45	0%	0–8.0%	-	-
Prior TB History	-	-	-	-	-
With Prior TB	1/2	50%	1.3–98.7%	No Prior TB	3.2%
No Prior TB	3/93	3.2%	0.7–9.2%	-	-
Residence	-	-	-	-	-
Urban	4/54	7.4%	2.0–17.5%	Rural	0%
Rural	0/41	0%	0–8.5%	-	-
Comorbidity Status	-	-	-	-	-
With Comorbidity	4/69	5.8%	1.6–14.0%	No Comorbidity	0%
No Comorbidity	0/26	0%	0–13.2%	-	-
Socioeconomic Status	-	-	-	-	-
Middle/High SES	4/58	6.9%	1.9–16.5%	Low SES	0%
Low SES	0/37	0%	0–9.5%	-	-

Effect sizes and clinical significance

Effect sizes were calculated to quantify the magnitude of observed differences independent of sample size. The largest effect size was observed for dialysis duration, with Cohen’s d=0.82, representing a large practical significance. The mean difference of 34.65 months between positive (3.75±2.87 months) and negative (38.4±30.1 months) cases demonstrates a clinically meaningful difference in the timing of LTBI detection relative to dialysis initiation. Monthly family income showed a medium effect size (Cohen’s d=0.52), with positive cases earning approximately ₹5,725 more monthly than negative cases (₹16,250 vs ₹10,525, respectively). Weight showed a small-to-medium effect size (Cohen’s d=0.40), while age demonstrated a negligible effect size (Cohen’s d=-0.15). Table 6 shows the effect sizes (Cohen’s d) for continuous variable comparisons.

**Table 6:** Effect Sizes (Cohen’s d) for Continuous Variable Comparisons Cohen’s d interpretation: <0.2 = negligible; 0.2-0.5 = small; 0.5-0.8 = medium; >0.8 = large.

Comparison	Positive Mean±SD	Negative Mean±SD	Mean Difference	Cohen’s d	Interpretation	Clinical Significance
Dialysis Duration	3.75±2.87	38.4±30.1	34.65 months	0.82	Large	Clinically meaningful
Monthly Income	₹16,250±6,904	₹10,525±12,418	₹5,725	0.52	Medium	Modest difference
Weight (kg)	59.0±8.2	54.5±10.2	4.5 kg	0.40	Small-Medium	Minor difference
Age (years)	50.0±11.4	51.9±13.1	1.9 years	-0.15	Negligible	Clinically trivial

## Discussion

This hospital‑based cross‑sectional study represents the first systematic evaluation of the Cy‑TB immunological test for LTBI detection in CKD patients undergoing maintenance hemodialysis. In this small single‑center cohort, Cy‑TB showed perfect agreement with IGRA among the four LTBI‑positive cases identified, suggesting high diagnostic concordance in this context; however, because IGRA testing was not performed in Cy‑TB‑negative participants, formal diagnostic accuracy measures (sensitivity, specificity, and predictive values) could not be determined. The overall LTBI prevalence of 4.2% (95% CI: 1.2%-10.3%) in our study population was substantially lower than the 15-25% range reported in prior international studies of dialysis patients, suggesting important geographic and population-specific variations in LTBI burden [17,18]. However, within the constraints of the small sample size and selective IGRA verification, the observation that dialysis duration <6 months was associated with a higher proportion of Cy‑TB‑positive cases (75% of positives in this phase) may indicate that the early dialysis phase is a period of relatively increased LTBI detection in this cohort and should be viewed as a hypothesis‑generating signal rather than a definitive change in our understanding of TB infection dynamics.

The higher proportion of Cy‑TB‑positive results observed in the early dialysis phase (<6 months) compared with later dialysis duration (17.6% versus 1.3% in our sample) is an interesting pattern that may indicate a period of relatively increased LTBI detection soon after dialysis initiation. However, this pattern is derived from only four LTBI‑positive cases and is therefore statistically fragile; even a single additional positive case in the ≥6‑month group would substantially change the estimated effect size and p‑values. Because the study is cross‑sectional, with very low event counts and selective IGRA verification, we cannot determine whether this apparent early‑dialysis signal reflects underlying biological changes, differences in exposure history, or random variation. Mechanistic explanations, including the possibility of partial immune recovery after initiation of dialysis, are biologically plausible but were not directly assessed and remain speculative [19,20]. Accordingly, the observed early‑dialysis association should be regarded as a preliminary, hypothesis‑generating finding that requires confirmation in larger, longitudinal studies with comprehensive parallel testing [8,17,21].

Prior history of TB appeared as a statistically significant risk factor for LTBI detection in this small cohort (p=0.048, OR=25.2, 95% CI: 1.4-442.8), with one of two patients with documented prior TB testing positive compared to three of 93 without prior TB history. However, this estimate is based on only two participants with a prior TB history and four LTBI‑positive cases overall, resulting in extremely wide confidence intervals and considerable statistical instability. As such, this association should be viewed as exploratory and hypothesis‑generating rather than definitive. While the absolute number of patients with prior TB was limited (n=2), the large odds ratio is consistent with the biologically plausible notion that previous TB disease may be associated with an increased likelihood of LTBI in dialysis patients. However, the very wide confidence intervals and small event numbers mean that our data cannot quantify this risk with precision or support specific preventive treatment strategies on their own; instead, they highlight the importance of systematically documenting prior TB history and evaluating its role in risk stratification in larger future studies [8].

Our study identified several variables that showed marginal trends toward association with LTBI positivity, but none of these reached conventional statistical significance, and all are based on very small numbers. Urban residence demonstrated a marginal trend (p=0.073), with all four positive cases residing in urban areas (7.4% prevalence in urban residents versus 0% in rural residents), which is compatible with the possibility of higher TB exposure in densely populated urban settings but cannot be confirmed from our data. The presence of any comorbid condition also showed a marginal association (p=0.072), with all four positive cases having at least one comorbidity compared to 67% of negative cases, but the low event count limits the reliability of this estimate. Similarly, the observation of a marginal association with recent fever (p=0.099; one positive case) may suggest subclinical immune activation but is too sparse to support any firm conclusions. These marginal findings should therefore be interpreted solely as exploratory signals to be examined in adequately powered studies [7,8].

In contrast to these marginal trends, numerous variables demonstrated no statistically significant association with LTBI positivity in our dataset. Age (p=0.854), gender (p=0.591), body weight (p=0.381), and BMI showed no significant associations, although these factors have been implicated as risk modifiers in other immunocompromised populations. Given the small number of LTBI‑positive cases, our study may simply lack power to detect modest effects of these variables, and we cannot distinguish a true absence of association from type II error. Similarly, smoking status (p=0.339) and alcohol consumption (p=0.861) showed no significant associations; this could reflect insufficient sample size, misclassification due to self‑report, or a genuinely attenuated effect in the context of severe uremia‑related immunosuppression. These null findings should therefore not be interpreted as evidence that these factors are unimportant, but rather as inconclusive results that require further investigation in larger cohorts [8,17].

Respiratory symptoms, including persistent cough (p=0.403), dyspnea (p=0.359), productive cough (p=0.359), and night sweats (p=0.172), showed no significant associations with Cy‑TB positivity, a finding that is consistent with current understanding of LTBI but should be interpreted cautiously given the small number of positive cases. The absence of a statistical association between respiratory symptoms and LTBI positivity highlights the well-recognized phenomenon that LTBI, by definition, causes no clinical manifestations and is not transmissible, rendering symptom assessment unhelpful for identifying individuals with LTBI in this population. Notably, all patients underwent structured symptom assessment to exclude active TB disease, and all enrolled patients were symptom-negative; the continued lack of association between specific symptoms and LTBI positivity in this symptom-screened cohort confirms the clinical appropriateness of our symptom-based exclusion strategy. TB contact history (p=0.572) and BCG vaccination status (p=0.511) also showed no significant associations with Cy-TB positivity; the null finding for TB contact history is somewhat unexpected and may reflect inadequate recall of TB exposures by study participants, particularly exposures occurring years or decades before dialysis initiation. The absence of an apparent association between BCG vaccination status and Cy‑TB positivity in this small cohort is consistent with the expected higher specificity of Cy‑TB compared to traditional tuberculin skin testing, but our study was not designed or powered to definitively compare the specificity between these tests [8,10,22].

Effect size analysis suggested that dialysis duration had the largest apparent clinical impact on LTBI detection (Cohen's d=0.82, representing large practical significance), with a mean difference of 34.65 months between positive and negative cases. This large effect size confirms that dialysis duration is not merely statistically significant but clinically meaningful in predicting LTBI status in this population. Monthly family income demonstrated a medium effect size (Cohen's d=0.52), with positive cases having approximately ₹5,725 higher monthly income than negative cases (₹16,250 versus ₹10,525), suggesting a possible socioeconomic gradient in TB exposure or healthcare-seeking behavior in our Indian study population [8]. Weight showed a small-to-medium effect size (Cohen's d=0.40), while age demonstrated a negligible effect size (Cohen's d=-0.15), confirming that demographic variables are substantially less important than dialysis-duration-related factors in determining LTBI status. However, these effect size estimates are drawn from only four LTBI‑positive cases and should therefore be interpreted with caution as approximate indicators of potential clinical relevance rather than precise measures.

The study population was predominantly male (60%, n=57) and middle-aged (mean 51.8±13.0 years), with a substantial comorbidity burden (68.4% with at least one comorbid condition). Hypertension was the predominant comorbidity (57.9%), reflecting the typical epidemiological pattern in dialysis populations, while diabetes mellitus affected 31.6% of participants. These demographic and clinical characteristics are representative of typical dialysis cohorts in India, supporting the generalizability of our findings to similar CKD populations in India and other countries with similar epidemiology [7,9].

Limitations

Our study was subject to several important limitations that should be considered when interpreting the findings. First, the overall LTBI prevalence of 4.2% was substantially lower than the 15-25% range reported in prior international studies, potentially reflecting geographic variation in TB burden, differences in LTBI diagnostic methodology, or the inclusion of newly dialysed patients with unknown LTBI status who may represent a healthier subset compared to chronic dialysis populations. The low overall prevalence resulted in only four Cy‑TB-positive cases across 95 patients, leading to extremely low event counts. This severely limits statistical power, yields very imprecise odds ratios and subgroup prevalence estimates (with very wide confidence intervals, e.g., prior TB history with n=2 total cases), increases the risk of spurious associations, and means that all regression and subgroup analyses should be interpreted as exploratory and hypothesis‑generating rather than definitive.

Second, this was a single‑center, hospital‑based cross‑sectional study with a relatively small sample size, which limits external generalizability to other dialysis populations and precludes causal inference; we cannot determine whether dialysis initiation itself predisposes to LTBI or whether newly dialysed patients simply represent a distinct temporal window of heightened TB susceptibility. Third, our study was conducted in a single tertiary care hospital hemodialysis unit and enrolled only patients on maintenance hemodialysis; findings may not apply to patients on peritoneal dialysis, who have different immunological profiles and potentially different TB risk, or to dialysis centers with different demographic characteristics, TB epidemiology, or healthcare infrastructure.

Fourth, participant selection through consecutive sampling from a single hemodialysis unit may have introduced selection bias if the enrolled cohort differed systematically from the broader dialysis population in terms of TB risk factors or health‑care‑seeking behavior. Fifth, symptom assessment relied on patient recall and self‑reporting, which is vulnerable to recall bias and social desirability bias, particularly for behavioral factors such as smoking and alcohol use. Sixth, mycobacterial culture, the gold standard for TB diagnosis, was not performed on sputum samples; however, all specimens were negative on AFB microscopy and CBNAAT, providing strong evidence that no study participants had active pulmonary tuberculosis. Seventh, IGRA was used as a reference test only in Cy‑TB-positive individuals; Cy‑TB-negative participants were not systematically verified with IGRA. This verification bias prevents estimation of sensitivity, specificity, and negative predictive value and means that we are reporting agreement between Cy‑TB and IGRA among positives rather than comprehensive diagnostic accuracy or robust positive and negative predictive values for the entire cohort. Eighth, Cy‑TB readings were performed by non‑blinded assessors, which may have introduced observer bias in delineating induration borders and measuring transverse diameters, although this risk was partly mitigated by adherence to standardized NTEP procedures and the use of objective millimeter measurements with a prespecified ≥5 mm cut‑off.

Despite these limitations, our study provides preliminary, hypothesis‑generating data suggesting that the early dialysis phase (<6 months from initiation) may represent a higher‑yield temporal window for LTBI detection in CKD‑dialysis patients; this signal requires confirmation in larger, prospective, comparative studies before any changes to routine screening protocols are recommended.

Future prospective studies

Future prospective studies are needed to characterize the immunological mechanisms underlying the surge in LTBI detection in early dialysis through longitudinal assessment of T-cell subsets, mycobacterial-specific immune responses, and uremic toxin clearance patterns; to establish the optimal timing, frequency, and cost-effectiveness of TB screening protocols in dialysispopulations; and to conduct comprehensive diagnostic accuracy studies comparing Cy-TB and IGRA across larger and more diverse hemodialysis cohorts to definitively establish the sensitivity, specificity, positive predictive value, and negative predictive value of Cy-TB in severe immunosuppression.

## Conclusions

This hospital‑based cross‑sectional study suggests that the Cy‑TB immunological test shows high agreement with the IGRA reference standard for detecting LTBI in maintenance hemodialysis patients, within the limits of a small single‑center cohort. The most notable observation is a higher LTBI detection rate during the early dialysis phase (within six months of treatment initiation) compared with later dialysis duration in this sample, which raises the possibility that early dialysis may represent a higher‑yield window for LTBI screening.

This novel finding challenges the conventional assumption of relatively stable LTBI prevalence across dialysis duration categories and suggests that immune reconstitution phenomena following the initiation of dialytic uremic toxin removal may transiently unmask previously suppressed mycobacterial-specific immune responses. Prior history of tuberculosis emerged as another significant predictor of LTBI positivity, with substantially elevated odds ratios indicating that patients with documented prior TB exposure warrant intensive preventive therapy strategies. Collectively, these findings suggest that dialysis duration <6 months and prior TB history may be useful risk‑stratification factors for prioritizing LTBI screening in CKD‑dialysis patients. However, given the very small number of LTBI‑positive cases, the single‑center cross‑sectional design, and the absence of universal IGRA testing, these observations should be regarded as preliminary and hypothesis‑generating. Larger, prospective, comparative studies are required to confirm these signals and to determine the true diagnostic performance and clinical impact of Cy‑TB before any changes to screening protocols are recommended.
